# Relationship between physical activity and locomotive syndrome among young and middle-aged Japanese workers

**DOI:** 10.1093/joccuh/uiae001

**Published:** 2024-01-08

**Authors:** Kazuhiko Watanabe, Xi Lu, Shota Masuda, Takeshi Miyamoto, Takahiko Katoh

**Affiliations:** Department of Public Health, Faculty of Life Sciences, Kumamoto University, Kumamoto, 860-8556, Japan; Division of Rehabilitation Technology, Department of Medical Technology, Kumamoto University Hospital, Kumamoto, 860-8556, Japan; Department of Public Health, Faculty of Life Sciences, Kumamoto University, Kumamoto, 860-8556, Japan; Department of Public Health, Faculty of Life Sciences, Kumamoto University, Kumamoto, 860-8556, Japan; Department of Orthopedic Surgery, Faculty of Life Sciences, Kumamoto University, Kumamoto, 860-8556, Japan; Department of Public Health, Faculty of Life Sciences, Kumamoto University, Kumamoto, 860-8556, Japan

**Keywords:** locomotive syndrome, physical activity, sedentary behavior, occupational, worker, healthy life expectancy

## Abstract

**Objectives:** This study aimed to examine the relationship between physical activity (PA) and locomotive syndrome (LS) among young and middle-aged Japanese workers.

**Methods:** This cross-sectional study included 335 participants from a company in Kumamoto, Japan. LS was evaluated using the 25-question Geriatric Locomotive Function Scale (GLFS-25); a GLFS-25 score ≥7 was defined as LS. Weekly PA was measured using the International Physical Activity Questionnaire. Work-related PA (time spent sitting, standing, walking, and strenuous work per day) and sedentary breaks were measured using a Work-related Physical Activity Questionnaire. Screen usage (television [TV], smartphones, tablets, and personal computers) during leisure time was recorded. The association between PA and LS was examined using a multivariate logistic regression analysis adjusted for age, sex, body mass index, history of musculoskeletal disorders, cancer, stroke, occupation, employment type, work time, shift system, employment status, and body pain.

**Results:** A total of 149 participants had LS. Fewer sedentary breaks during work (>70-minute intervals, odds ratio [OR] = 2.96; prolonged sitting, OR = 4.12) and longer TV viewing time (≥180 minutes, OR = 3.02) were significantly associated with LS. In contrast, moderate PA (OR = 0.75) was significantly associated with a lower risk of LS.

**Conclusions:** Fewer sedentary breaks during work and longer TV viewing time could increase the risk of LS in young and middle-aged Japanese workers.

## Key points

**What is already known on this topic:** Moderate-intensity physical activity (PA) is associated with a low risk of locomotive syndrome (LS) in elderly women; however, the relationship between PA intensity, sedentary behavior, and LS in young and middle-aged individuals is unknown.

**What this study adds:** Moderate-intensity PA was associated with a low risk of LS, whereas sedentary behavior such as long television viewing time and few sedentary breaks during work was associated with LS among young and middle-aged workers.

**How this study might affect research, practice, or policy:** As a countermeasure against LS among young and middle-aged workers, strategies to reduce sedentary time during leisure and work time are worth exploring.

## 1. Introduction

Japan has the highest life expectancy globally, and the population of older adults aged ≥65 years was 36.19 million (28.8%) in 2020.^1^ By 2065, 1 in 2.6 people will be aged 65 years or older, and 1 in 3.9 will be aged 75 years or older.^1^ However, there has been no significant change in this century, with a 9-year gap between average and healthy life expectancies in Japan.^1^ Japan currently has 6.69 million elderly individuals requiring nursing care services, resulting in substantial costs, and urgent measures to promote healthy life expectancy are needed.^2^ Notably, functional decline from musculoskeletal issues, such as joint disease (10.2%) and fracture/fall (13.9%), are major factors.^3^ Therefore, promoting healthy life expectancy in terms of motor dysfunction and mobility among young and middle-aged individuals is crucial.

In Japan, the retirement age has been extended from 65 to 70 years after a 2021 law revision.^4^ The number of employed persons aged ≥65 years reached 9.12 million, with an employment rate of 25.2% in 2022.^5^ Moreover, older adults in Japan have a strong desire to work.^1^ Thus, younger working generations desire to maintain healthy lifestyles even after reaching the age of 65 years, emphasizing the importance of maintaining motor function in those aged <65 years as part of promoting the health of elderly workers.

The Japanese Orthopedic Association (JOA) defines locomotive syndrome (LS) as an early-stage indicator of mobility impairment due to locomotor issues.^6^^,^^7^ LS, though reversible and treatable, affects long-term care services in Japan.^8^^,^^9^ LS affects not only elderly individuals but also those aged <60 years.^9^^,^^10^ Recognizing motor dysfunctions such as LS among young and middle-aged individuals is vital for maintaining motor function and health in elderly workers. Epidemiological studies and methods for preventing and improving LS among young and middle-aged individuals are limited.^11^ Simple, widely understood LS indicators could raise awareness and promote regular action.

Reduced physical activity (PA) and increased sedentary habits, such as television (TV) viewing and prolonged sitting, pose mobility risks for middle-aged and elderly individuals.^12^ Sedentary lifestyles have been associated with poorer physical function, such as walking speed and instrumental activities of daily living score.^13^ Furthermore, moderate PA (≥3.0 metabolic equivalents [METs]) was associated with a decreased risk of LS in community-dwelling elderly women.^14^ Therefore, evaluating PA, including sedentary behavior, is crucial for improving mobility function and health in middle-aged and elderly individuals. Office workers who sat during working hours also sat for longer periods outside work.^15^ Thus, work-related sitting, occupying most of the day, could impact the health of long-term workers, emphasizing the need to consider PA both at and off work.

Exploring the association between PA intensity and LS in the workplace might help prevent LS and improve motor function. Notably, decreasing sedentary behavior could be a versatile approach for improving LS. However, the relationship between PA intensity, sedentary behavior, and LS in young and middle-aged individuals remains unclear around the world. This study aimed to investigate the association between PA, including sedentary behavior during work and leisure time, and LS among young and middle-aged Japanese workers.

## 2. Methods

### 2.1. Study design and participants

This cross-sectional study included white- and blue-collar workers aged 21-66 years from a pharmaceutical manufacturing company in Kumamoto City, Japan, who underwent regular health check-ups at the workplace by a physician and completed a self-administered questionnaire that encompassed PA-related behavior, body pain, medical history of musculoskeletal disorders, stroke, cancer, and asthma, lifestyle-related information, work-related information, and sociodemographic data. Data administrators distributed web- or paper-based self-administered questionnaires to willing participants and collected them during November and December 2022. Health check-ups included a questionnaire (comprising the medical history of diabetes, cardiovascular disease, hypertension, dyslipidemia, and anemia), physical examination, and laboratory tests.

Exclusion criteria included age ≤19 years, inability to walk independently, no longer employed, injury within 3 months from examination date and undergoing treatment, and failure to provide consent. No one met these conditions except for cases where consent was not obtained. We ensured complete data for LS stage diagnosis and other variables. All participants provided written informed consent.

### 2.2. Measurements

#### 2.2.1. Locomotive syndrome

LS was assessed using the 25-question Geriatric Locomotive Function Scale (GLFS-25).^16^ GLFS-25 is a comprehensive and self-administered tool for early detection of LS, with proven reliability and validity. It comprises 25 items regarding pain, activities of daily living, social functions, and mental health status during the last month. Each question is rated on a 5-point scale from no impairment (0 points) to severe impairment (4 points), and a higher total score (minimum 0, maximum 100) indicates poor locomotive function.^16^ The GLFS-25 is a clinically useful tool for assessing mobility in patients with LS.^17^ LS was defined as a total GLFS-25 score ≥7, and LS stage was classified based on JOA criteria^18^ as follows: LS stage 1: 7-15; LS stage 2: 16-23; and LS stage 3: ≥24. These stages represent the onset of mobility decline, its progression, and a significant decline disrupting social participation, respectively.^9^^,^^18^

#### 2.2.2. PA-related behavior

We assessed daily screen times for smartphones, tablets, and personal computers (PCs) outside work and TV viewing during leisure. Screen time was determined by the question, “How long do you use smartphones, tablets, and PCs, and watch television, outside work per day in a usual week?” Participants reported time spent using smartphones, tablets, and PCs, and TV viewing time separately. Screen time was divided into 4 categories based on the quartiles. Self-reported walking (W-PA), moderate-intensity PA (M-PA), vigorous-intensity PA (V-PA), and sedentary time on holidays were evaluated using a reliable and validated (Japanese version) short-form International Physical Activity Questionnaire (IPAQ)^19^^,^^20^ to determine average weekly PA. Total METs-minutes per week for W-PA, M-PA, and V-PA were calculated by multiplying 3.3, 4.0, and 8.0, respectively, by the daily or weekly time spent (minutes).^19^^,^^20^ We used the Work-related Physical Activity Questionnaire (WPAQ)^21^ to collect data on work: time spent sitting, standing, walking, daily strenuous work, and sedentary breaks. The WPAQ is a self-reported questionnaire with established reliability and criterion validity, including sedentary behavior and breaks.^21^ For work-related PA, the average sitting, standing, walking, and strenuous work times were calculated by multiplying their daily ratios and average daily work time. Sedentary breaks during work were determined by the question, “How often do you stand up while working in a sitting position?” Participants selected answers from 10 categories ranging from 0 to more than 90-minute intervals.^21^ We added the sentence, “Please select either ‘keep standing’ or ‘keep sitting’ if you never sit down or stand up during work outside break” separate from the WPAQ in the sedentary break questionnaire. If they selected “0” or “keep standing,” it was considered “keep standing during work time.” Sedentary breaks were divided into 4 categories: “keep standing or at 10-30-minute intervals,” “40-60-minute intervals,” “more than 70-minute intervals,” and “keep sitting.” Muscle-strengthening activity in this study was defined based on the World Health Organization PA guidelines^22^ as an exercise that increases skeletal muscle strength, induces breathlessness and fatigue, and involves muscles such as those in the arms, legs, and abdomen. In this study, daily screen times for smartphones, tablets, and PCs outside work, TV viewing during leisure, and sedentary time on holidays were defined as sedentary behavior during leisure time.

#### 2.2.3. Physical examination and laboratory measurements

Participants underwent a physical examination to assess height, body weight, body mass index (BMI), abdominal circumference, and systolic and diastolic blood pressures (SBP and DBP). BMI was calculated as body weight (kg) divided by height squared (m^2^) and classified as underweight (<18.5), normal weight (18.5-24.9), or overweight (≥25.0).^23^ Abdominal circumference was measured during normal expiration while standing. Data on body pain and medical history of musculoskeletal disorders, stroke, cancer, chronic obstructive pulmonary disease (COPD), asthma, diabetes, cardiovascular disease, hypertension, dyslipidemia, and anemia were collected. Body pain was defined as current pain in the back, lower back, hip joint, or knee joint^24^ as assessed by the question, “Do you currently experience pain in any of these areas?” Musculoskeletal disorders included osteoporosis, vertebral fracture, scoliosis, lower-limb fracture, hip osteoarthritis, knee osteoarthritis, spinal canal stenosis, herniated disks, rheumatoid arthritis, and other disorders or symptoms. Fasting blood samples were obtained during routine health check-ups to measure serum levels of fasting glucose, γ-glutamyl transpeptidase, total cholesterol (TC), aspartate aminotransferase, alanine aminotransferase, high-density lipoprotein cholesterol (HDL-C), low-density lipoprotein cholesterol (LDL-C), triglyceride (TG), hemoglobin, white blood cell count, and red blood cell count.

#### 2.2.4. Lifestyle-related information

Cohabitation was determined by the question, “Do you currently live with anyone?” Smoking status was categorized as never smoked (never or smoked <10 cigarettes daily for <1 year), former smoker (not currently smoking), or current smoker (smoked at the time of examination). Pack-years were calculated by averaging daily cigarette, e-cigarette, and heated cigarette use, dividing by 20, and multiplying by years smoked. Alcohol consumption categories included nondrinking, 1-6 d/wk, and daily drinking.^23^ Dietary habits were assessed by meal frequency (3 meals/d). Sleep duration was evaluated as daily or weekly sleeping hours.

#### 2.2.5. Work-related information

Data on employment type, job duration, occupation, employment status, shift system, and daily work duration were obtained. Employment types included regular staff, entrusted employees, part-time workers, and others. Job duration was classified as full-time or part-time based on daily work hours because some part-time workers may actually work full-time based on employment type. Participants were divided into blue-collar (manufacturing, security, carrying, and cleaning) and white-collar (administrative and managerial, professional and engineering, clerical, and sales) workers. Employment status was classified as manager, foreman, or another. The presence of a shift system after 10:00 pm. was assessed. Daily work time was evaluated using the WPAQ.^21^

#### 2.2.6. Sociodemographic data

Data regarding age, sex, confidence in exercising, and interest in health were collected using a self-administered questionnaire. Confidence in exercise was assessed using the question, “Are you confident about exercising (No matter where, exercise type, and intensity)?” The answers “very unconfident” and “little confidence” were defined as unconfident, and the answers “somewhat confident” and “very confident” were defined as “confident.” Interest in health was assessed using the question “Are you interested in your health?” with 4 answer choices of “not at all,” “little,” “somewhat interested,” and “very interested”; the former 2 answers were defined as “not interested,” and the latter 2 were defined as “interested.”

### 2.3. Statistical analyses

Data distribution normality was assessed using the Shapiro-Wilk test. Student’s *t*-test and the Mann-Whitney *U* test were performed to examine the differences in normally and nonnormally distributed continuous variables, respectively, between participants with and without LS. The chi-square test was used to assess the differences in categorical variables between the 2 groups.

Odds ratios (ORs) and 95% CIs were used to evaluate the associations between LS and PA-related behavior. Unadjusted models were used to assess the relationships between each variable and LS. Multivariate logistic regression models were used to control for potential confounding factors. Weekly strenuous work time, W-PA, M-PA, and V-PA (METs/wk) were nonnormally distributed and log-transformed for multiple logistic regression analysis. PA during working hours and weekly PA were used as continuous data. ORs for LS were calculated for PA-related behavior with body pain, adjusting for age, sex, and BMI (Model 1). The ORs were calculated after adjusting for history of musculoskeletal disorders (osteoporosis, vertebral fracture, scoliosis, lower limb fracture, knee osteoarthritis, spinal canal stenosis, herniated disk, rheumatoid arthritis, and other musculoskeletal disorders or symptoms), cancer, and stroke (Model 2). Further adjustments were made for occupation, employment type, work time, shift system, and employment status (Model 3). Shorter sedentary time was used as the reference category for each independent PA-related variable. Clinically related and significant variables were used for multivariate analyses after confirming multicollinearity among the variables.

All statistical analyses were performed using SPSS version 22 (IBM Corp., Armonk, NY, USA) based on a 2-tailed probability. Statistical significance was set at *P* < .05. No data were missing in the final analysis. The minimum required sample size for this study, determined by power analysis (G*power, Version 3.1, sample size calculation for logistic regression analysis), was 119 in each group.

### 2.4. Ethics approval

This study was conducted in accordance with the Declaration of Helsinki and the Ethical Guidelines for Epidemiological Research. The Human Ethics Committee of Kumamoto University approved the study (Number 2527).

## 3. Results

### 3.1. Study participants’ characteristics

**Figure 1:**
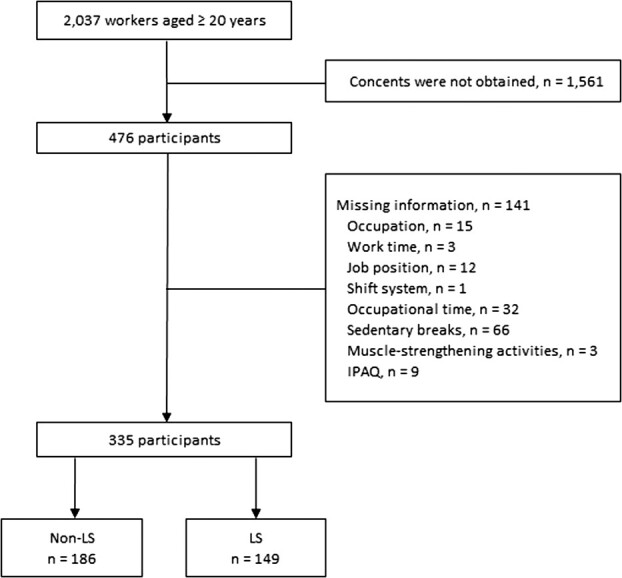
Flowchart for study participant selection.

Among 2037 participants, 476 returned their questionnaires. Participants lacking data on occupation (*n* = 15), work time (*n* = 3), job position (*n* = 12), shift system (*n* = 1), occupational time (*n* = 32), sedentary breaks (*n* = 66), muscle-strengthening activities (*n* = 3), or IPAQ^19^ (*n* = 9) were excluded. After excluding 141 participants, 335 participants were included in the analysis (Figure 1). Table 1 presents the baseline characteristics of the participants according to age, sex, physical examination, and laboratory measurements. The mean age of participants was 44.2 years, 57.6% were females, mean BMI was 21.7, and 73.1% had normal weight. The prevalence of LS was 44.5% (Table 1).

**Table 1:** Participants’ characteristics according to LS.

	**Total** ***n* = 335**	**Non-LS** ***n* = 186**	**LS** ***n* = 149**	***P* value**
**Age**	44.2 (11.7)	43.1 (11.9)	45.1 (11.4)	.202
**Female,** ^**a**^ ***n* (%)**	193 (57.6)	99 (53.2)	94 (63.1)	.070
**Height, cm**	164.1 (8.6)	164.6 (8.7)	163.4 (8.4)	.201
**Body weight, kg**	58.7 (11.3)	58.5 (10.7)	59.1 (12.1)	.635
**BMI, kg/m** ^**2**^	21.7 (3.2)	21.5 (2.8)	22.0 (3.6)	.129
**Underweight,** ^**a**^ ***n* (%)**	47 (14.0)	24 (12.9)	23 (15.4)	
**Normal,** ^**a**^ ***n* (%)**	245 (73.1)	144 (77.4)	101 (67.8)	
**Overweight,** ^**a**^ ***n* (%)**	43 (12.8)	18 (9.7)	25 (16.8)	.096
**Abdominal circumference, cm**	78.5 (9.2)	77.4 (8.3)	79.8 (10.1)	**.020**
**SBP, mmHg**	115.6 (12.4)	115.0 (11.7)	116.3 (13.3)	.360
**DBP, mmHg**	71.0 (9.9)	70.6 (9.4)	71.4 (10.7)	.484
**Body pain (back, low back, hip, or knee),** ^**a**^ ***n* (%)**	139 (41.5)	55 (29.6)	84 (56.4)	**<.001**
**Medical history,** ^**a**^ ***n* (%)**				
**Musculoskeletal disorders**	74 (22.1)	27 (14.5)	47 (31.5)	**<.001**
**Osteoporosis**	10 (3.0)	3 (1.6)	7 (4.7)	.099
**Vertebral fracture**	3 (0.9)	0 (0.0)	3 (2.0)	.052
**Scoliosis**	11 (3.3)	3 (1.6)	8 (5.4)	.055
**Lower limb fracture**	12 (3.6)	4 (2.2)	8 (5.4)	.115
**Knee osteoarthritis**	6 (1.8)	1 (0.5)	5 (3.4)	.053
**Spinal canal stenosis**	5 (1.5)	0 (0.0)	5 (3.4)	**.012**
**Herniated disk**	37 (11.0)	17 (9.1)	20 (13.4)	.214
**Rheumatoid arthritis**	2 (0.6)	0 (0.0)	2 (1.3)	.113
**Other musculoskeletal disorders or symptoms**	16 (4.8)	4 (2.2)	12 (8.1)	**.012**
**Stroke**	2 (0.6)	2 (1.1)	0 (0.0)	.204
**Cancer**	12 (3.6)	5 (2.7)	7 (4.7)	.204
**Asthma**	29 (8.7)	19 (10.2)	10 (6.7)	.257
**Diabetes**	3 (0.9)	2 (1.1)	1 (0.7)	.696
**Cardiovascular disease**	6 (1.8)	2 (1.1)	4 (2.7)	.370
**Hypertension**	66 (19.7)	31 (16.7)	35 (23.5)	.119
**Dyslipidemia**	21 (6.3)	7 (3.8)	14 (9.4)	**.035**
**Anemia**	53 (15.8)	24 (12.9)	29 (19.5)	.102
**Laboratory data**				
**Fasting glucose, mg/dL**	92.7 (10.3)	92.4 (10.4)	93.0 (10.2)	.602
**γ-GTP, IU/L**	29.6 (38.2)	32.0 (42.7)	26.7 (31.5)	.205
**TC, mg/dL**	203.8 (32.6)	199.7 (31.4)	208.6 (33.5)	.084
**ALT,**^**b**^ **IU/L**	19.6 (14.1)	16.0 (10.5-21.5)	16.0 (11.0-21.0)	.594
**AST, IU/L**	20.2 (6.4)	19.7 (6.0)	20.8 (7.0)	.117
**HDL-C, mg/dL**	72.4 (17.6)	71.4 (17.0)	73.6 (18.2)	.249
**LDL-C, mg/dL**	115.0 (28.9)	113.6 (28.6)	116.8 (29.3)	.316
**TG, mg/dL**	84.6 (96.2)	89.8 (121.5)	78.0 (48.3)	.265
**Hb, g/dL**	13.8 (1.3)	13.9 (1.3)	13.7 (1.2)	.143
**RBC, ×10**^**4**^**/μL**	456.1 (40.5)	457.6 (40.0)	453.8 (41.1)	.353
**WBC, *n*/μL**	5172.8 (1332.1)	5174.7 (1341.5)	5170.4 (1324.9)	.977

Data are presented as means (SD), median (interquartile range), or as *n* (percentage). Bold P values denote statistical significance with less than 0.05. ALT, alanine aminotransferase; AST, aspartate aminotransferase; BMI, body mass index; DBP, diastolic blood pressure; Hb, hemoglobin; HDL-C, high-density lipoprotein cholesterol; LDL-C, low-density lipoprotein cholesterol; LS, locomotive syndrome; RBC, red blood cells; SBP, systolic blood pressure; TC, total cholesterol; TG, triglyceride; WBC, white blood cells; γ-GTP, γ-glutamyl transpeptidase.

a Examined by the chi-square test.

b Examined by the Mann-Whitney *U* test.

There were no significant differences in age, sex, height, body weight, BMI, SBP, or DBP between the 2 groups. However, participants with LS had larger abdominal circumference (*P* = .020) and a higher likelihood of body pain (*P* < .001). Moreover, the LS group had a higher number of spinal canal stenoses (*P* = .012) and other musculoskeletal disorders or symptoms (*P* = .012). There were no cases of diagnosed hip osteoarthritis or COPD. Whereas those in the LS group had more medical histories of dyslipidemia than those without LS (*P* = .035), blood parameters (TC, LDL-C, and TG) did not significantly differ between the 2 groups. Medical history of stroke, cancer, asthma, diabetes, cardiovascular disease, hypertension, and anemia did not significantly differ between participants with and without LS.

The prevalence of LS across different age groups is presented in Table S1, ranging from 10.7% to 28.9% in each age group. Those aged 30-59 years accounted for 75% of LS cases, with no significant difference between each age group.

Table 2 outlines baseline characteristics of lifestyle factors, work-related information, confidence in exercising, and personal interest in health. No differences in cohabitation, smoking status, alcohol consumption, dietary habits, and sleep duration were observed among the groups. In the LS group, there were fewer regular staff and managers (*P* = .020 and .029, respectively), and the mean daily work time was shorter compared with that of those without LS (*P* = .006). No differences in occupation and number of shift systems were observed among the groups. Additionally, the number of individuals with confidence in exercising and an interest in their health did not differ between the 2 groups.

**Table 2:** Participants’ lifestyle characteristics and work-related information.

	**Total** ***n* = 335**	**Non-LS** ***n* = 186**	**LS** ***n* = 149**	***P* value**
**Living together,** ^**a**^ ***n* (%)**	285 (85.1)	158 (84.9)	127 (85.2)	.941
**Smoking status,** ^**a**^ ***n* (%)**				
**Never smoked**	246 (73.4)	137 (73.7)	109 (73.2)	
**Former smoker**	68 (20.3)	37 (19.9)	31 (20.8)	
**Current smoker**	21 (6.3)	12 (6.4)	9 (6.0)	.147
**Pack-years**^**b**^	0.0 (0.0-0.1)	0.0 (0.0-0.2)	0.0 (0.0-0.2)	.975
**Alcohol consumption,** ^**a**^ ***n* (%)**				
**None**	143 (42.7)	73 (39.3)	70 (47.0)	
**1-6 d/wk**	148 (44.2)	91 (48.9)	57 (38.3)	
**Daily**	44 (13.1)	22 (11.8)	22 (14.7)	.147
**Dietary habit,** ^**b**^ **d/wk**	5.7 (2.2)	5.8 (2.1)	5.7 (2.2)	.236
**Daily,**^**a**^ ***n* (%)**	216 (64.4)	122 (65.6)	94 (63.1)	
**1-6 d/wk, *n* (%)**^**a**^	99 (29.6)	55 (29.6)	44 (29.5)	
**None,**^**a**^ ***n* (%)**	20 (6.0)	9 (4.8)	11 (7.4)	.613
**Sleep duration,** ^**b**^ **min/d**	390.0 (360.0-420.0)	390.0 (360.0-420.0)	390.0 (360.0-420.0)	.328
**Confident about exercising,** ^**a**^ ***n* (%)**	220 (65.7)	122 (65.6)	98 (65.8)	.972
**Interest in their health,** ^**a**^ ***n* (%)**	307 (91.6)	169 (90.9)	138 (92.6)	.564
**Type of employment,** ^**a**^ ***n* (%)**				
**Regular staff**	209 (62.4)	128 (68.8)	81 (54.4)	
**Entrusted employee**	17 (5.1)	7 (3.8)	10 (6.7)	
**Part-time worker**	107 (31.9)	49 (26.3)	58 (38.9)	
**Other**	2 (0.6)	2 (1.1)	0 (0.0)	**.020**
**Job time,** ^**a**^ ***n* (%)**				
**Full-time**	239 (71.3)	140 (75.3)	99 (66.4)	
**Part-time**	96 (28.7)	46 (24.7)	50 (33.6)	.076
**Occupation,** ^**a**^ ***n* (%)**				
**White collar**	222 (66.3)	124 (66.7)	98 (65.8)	
**Blue collar**	113 (33.7)	62 (33.3)	51 (34.2)	.863
**Employment status,** ^**a**^ ***n* (%)**				
**Manager**	66 (19.7)	45 (24.2)	21 (14.1)	
**Foreman**	35 (10.4)	22 (11.8)	13 (8.7)	
**Other**	234 (69.9)	119 (64.0)	115 (77.2)	**.029**
**Shift system,** ^**a**^ ***n* (%)**	2 (0.6)	1 (0.5)	1 (0.7)	.875
**Daily work time, min/d**	498.9 (88.8)	510.8 (88.9)	484.0 (86.7)	**.006**

Data are presented as means (SD), median (interquartile range), or *n* (percentage). Bold P values denote statistical significance with less than 0.05. Dietary habit was defined as frequency of 3 meals per day during a week. LS, locomotive syndrome.

a Examined by the chi-square test.

b Examined by the Mann-Whitney *U* test

Table 3 presents various PA-related behaviors. Strenuous work time differed significantly between the 2 groups, whereas standing time, sitting time, and sedentary breaks during work time did not. Weekly M-PA was shorter in the LS group compared with those without LS (*P* = .008). However, TV viewing time during leisure and sedentary time on holidays were longer in the LS group (*P* = .002 and .032, respectively). Screen time for smartphones, tablets, and PCs and the number of participants engaged in muscle-strengthening activities showed no significant differences between the 2 groups.

**Table 3:** Physical activity-related behavior, according to LS.

**Variables**	**Total** ***n* = 335**	**Non-LS** ***n* = 186**	**LS** ***n* = 149**	***P* value**
**Work-related physical activity, min/d**				
**Sitting time at work**	338.7 (153.5)	345.6 (149.3)	330.1 (158.7)	.360
**Standing time at work**	77.7 (73.4)	83.2 (77.1)	70.9 (68.1)	.126
**Walking time at work**	71.3 (51.4)	73.3 (49.9)	68.7 (53.3)	.419
**Strenuous work time**^**a**^	0.0 (0.0-18.0)	0.0 (6.5)	0.0 (22.5)	**.038**
**Sedentary breaks during work time,** ^**a**^ ***n* (%)**				
**Keep standing at 10-30-min intervals**	142 (42.4)	83 (44.6)	59 (39.6)	
**At 40-60-min intervals**	155 (46.3)	86 (46.2)	69 (46.3)	
**More than 70-min intervals**	25 (7.4)	12 (6.5)	13 (8.7)	
**Keep sitting**	13 (3.9)	5 (2.7)	8 (5.4)	.458
**Weekly physical activity, METs/wk**				
**Walking**^**b**^	297.0 (0.0-693.0)	297.0 (0.0-693.0)	297.0 (0.0-693.0)	.710
**Moderate-intensity**^**b**^	0.0 (0.0-240.0)	0.0 (0.0-240.0)	0.0 (0.0-0.0)	**.008**
**Vigorous-intensity**^**b**^	0.0 (0.0-0.0)	0.0 (0.0-0.0)	0.0 (0.0-0.0)	.702
**Total (walk, moderate and vigorous intensity)**^**b**^	1110.6 (1517.7)	1242.5 (1683.2)	945.9 (1267.8)	.067
**Engage in muscle-strengthening activities,** ^**a**^ ***n* (%)**	149 (44.5)	84 (45.1)	65 (43.6)	.778
**Exercise days per week**	3.8 (2.1)	4.0 (2.1)	3.5 (2.0)	.119
**TV viewing time, min/d**	113.6 (82.4)	100.7 (74.1)	129.7 (89.3)	**.002**
**Quartile 1 (<60),**^**a**^ ***n* (%)**	62 (18.5)	41 (22.1)	21 (14.1)	
**Quartile 2 (60-119),**^**a**^ ***n* (%)**	99 (29.5)	59 (31.7)	40 (26.8)	
**Quartile 3 (120-179),**^**a**^ ***n* (%)**	83 (24.8)	48 (25.8)	35 (23.5)	
**Quartile 4 (≥180),**^**a**^ ***n* (%)**	91 (27.2)	38 (20.4)	53 (35.6)	**.014**
**Smartphone, tablet, and PC use time, min/d**	253. 3 (203.9)	233.9 (189.9)	277.6 (218.3)	.055
**Quartile 1 (<45),**^**a**^ ***n* (%)**	23 (6.9)	11 (5.9)	12 (8.1)	
**Quartile 2 (45-179),**^**a**^ ***n* (%)**	116 (34.6)	70 (37.6)	46 (30.9)	
**Quartile 3 (180-314),**^**a**^ ***n* (%)**	96 (28.7)	55 (29.6)	41 (27.5)	
**Quartile 4 (≥315),**^**a**^ ***n* (%)**	100 (29.8)	50 (26.9)	50 (33.5)	.392
**Sedentary time on weekdays, min/d**	376.8 (264.7)	363.4 (263.6)	393.6 (265.9)	.301
**Sedentary time on holidays, min/d**	370.5 (206.3)	348.9 (196.5)	397.5 (215.6)	**.032**

Data are presented as means (SD), median (interquartile range), or as *n* (percentage). Bold P values denote statistical significance with less than 0.05. LS, locomotive syndrome; METs, metabolic equivalents; PC, personal computer; TV, television.

a Examined by the chi-square test.

b Examined by the Mann-Whitney *U* test.

### 3.2. Association between PA-related behavior and LS

Univariate logistic regression analysis revealed that strenuous work time (crude OR, 1.01), TV viewing time (quartile 4 [≥180 minutes]; crude OR, 2.72), the presence of body pain (crude OR, 3.08), and M-PA (crude OR, 0.79) were significantly associated with LS. In multivariate logistic regression analyses adjusted for age, sex, BMI, medical history of musculoskeletal disorders, cancer, and stroke, occupation, employment type, work time, shift system, and employment status, as well as for the covariables of interest, M-PA (adjusted OR, 0.75) was associated with a lower risk of LS. Conversely, the presence of body pain (adjusted OR, 2.40), fewer sedentary breaks during work time (≥70-, 80-, and 90-minute intervals, keep sitting) (adjusted OR, 2.96 and 4.12, respectively), and longer TV viewing time (quartile 4 [≥180 minutes]; adjusted OR, 3.02) were associated with a higher risk of LS (Table 4). These associations were unaffected by age, sex, BMI, and other PA-related behaviors.

**Table 4:** Bivariate and multivariate associations of physical activity-related behavior, body pain, and LS.

		**Crude model**			**Model 1**			**Model 2**			**Model 3**		
**Variables**	**Number of cases** ^**(a)**^	**OR**	**95% CI**	***P* value**	**OR**	**95% CI**	***P* value**	**OR**	**95% CI**	***P* value**	**OR**	**95% CI**	***P* value**
**Physical activity during work time**													
**Sitting time per day**	335	1.00	1.00-1.00	.366	1.00	1.00-1.00	.834	1.00	1.00-1.00	.637	1.00	1.00-1.00	.778
**Standing time per day**	335	1.00	1.00-1.00	.128	1.00	1.00-1.16	.068	1.00	0.99-1.00	.195	1.00	0.99-1.00	.258
**Walking time per day**	335	1.00	0.99-1.00	.418	1.00	0.99-1.16	.248	1.00	0.99-1.00	.548	1.00	0.99-1.01	.540
**Strenuous work time per day**	335	**1.01** ^*^	1.00-1.02	**.030**	1.23	0.91-1.76	.157	1.41	1.00-1.98	.052	1.44	0.87-2.39	.157
**Sedentary breaks during work time**													
**Keep standing, 10-30-min intervals**	142	Ref.	—	—	Ref.	—	—	Ref.	—	—	Ref.	—	—
**40-60-min intervals**	155	1.13	0.71-1.79	.606	1.25	0.78-2.02	.357	1.35	0.81-2.25	.244	1.58	0.86-2.90	.141
**70, 80, ≥90-min intervals**	25	1.52	0.65-3.58	.333	1.71	0.72-4.08	.227	1.86	0.74-4.62	.185	**2.96** ^*^	1.01-8.68	**.048**
**Keep sitting**	13	2.25	0.70-7.22	.173	2.49	0.76-8.22	.113	2.55	0.74-8.78	.139	**4.12** ^*^	1.04-16.31	**.044**
**Screen time (TV viewing time)**													
**Quartile 1 (< 60 min)**	62	Ref.	—	—	Ref.	—	—	Ref.	—	—	Ref.	—	—
**Quartile 2 (60-119 min)**	99	1.32	0.68-2.57	.406	1.31	0.67-2.56	.438	1.50	0.74-3.04	.258	1.60	0.71-3.59	.257
**Quartile 3 (120-179 min)**	83	1.42	0.72-2.82	.311	1.33	0.66-2.69	.428	1.51	0.72-3.14	.273	1.78	0.75-4.18	.190
**Quartile 4 (≥180 min)**	91	**2.72** ^**^	1.39-5.33	**.003**	**2.51** ^*^	1.24-5.05	**.010**	**2.50** ^*^	1.19-5.26	**.016**	**3.02** ^*^	1.28-7.11	**.011**
**Screen time (smartphone, tablet, PC)**													
**Quartile 1 (<45 min)**	23	Ref.	—	—	Ref.	—	—	Ref.	—	—	Ref.	—	—
**Quartile 2 (45-179 min)**	116	0.60	0.25-1.48	.269	0.66	0.26-1.64	.367	0.76	0.28-2.03	.583	0.80	0.26-2.42	.687
**Quartile 3 (180-314 min)**	96	0.68	0.27-1.70	.413	0.83	0.32-2.17	.700	0.84	0.30-2.37	.744	1.07	0.32-3.52	.913
**Quartile 4 (≥315 min)**	100	0.92	0.37-2.27	.851	1.16	0.45-2.99	.764	1.24	0.45-3.45	.680	1.51	0.47-4.83	.489
**Pain (back, lower back, hip joint, knee joint)**													
**None**	196	Ref.	—	—	Ref.	—	—	Ref.	—	—	Ref.	—	—
**Presence**	139	**3.08** ^***^	1.96-4.84	**<.001**	**3.01** ^***^	1.89-4.80	**<.001**	**2.67** ^***^	1.64-4.35	**<.001**	**2.40** ^**^	1.39-4.14	**.002**
**Sedentary time on holiday**													
**Quartile 1 (<180 min)**	29	Ref.	—	—	Ref.	—	—	Ref.	—	—	Ref.	—	—
**Quartile 2 (180-299 min)**	90	1.10	0.46-2.65	.831	1.04	0.43-2.55	.925	0.98	0.39-2.46	.962	0.66	0.23-1.92	.434
**Quartile 3 (300-419 min)**	98	1.98	0.84-4.69	.121	1.84	0.77-4.40	.174	1.50	0.61-3.72	.382	0.83	0.29-2.35	.727
**Quartile 4 (≥420 min)**	118	1.72	0.74-4.00	.211	1.76	0.75-4.15	.196	1.45	0.59-3.54	.415	0.94	0.33-2.66	.884
**Engaged in muscle-strengthening activities**													
**Yes**	149	Ref.	—	—	Ref.	—	—	Ref.	—	—	Ref.	—	—
**No**	186	1.06	0.69-1.64	.778	1.08	0.69-1.69	.739	1.20	0.74-1.92	.461	1.26	0.70-2.28	.441
**Physical activity over a week**													
**Walk, log METs/wk**	335	0.96	0.82-1.13	.649	0.95	0.81-1.12	.547	0.92	0.77-1.08	.306	0.93	0.76-1.13	.469
**Moderate-intensity, log METs/wk**	335	**0.79** ^**^	0.66-0.94	**.008**	**0.81** ^*^	0.67-0.97	**.020**	**0.81** ^*^	0.67-0.98	**.032**	**0.75** ^*^	0.60-0.94	**.013**
**Vigorous-intensity, log METs/wk**	335	0.97	0.81-1.16	.711	1.02	0.85-1.24	.809	1.10	0.90-1.34	.349	1.21	0.95-1.54	.130

**Notes:**  ^*^ indicates p < 0.05; ^**^ indicates p < 0.01; ^***^ indicates p < 0.001. Bold P values denote statistical significance with less than 0.05. a. None of participants had missing data.

**Model 1**: age, sex, and BMI. **Model 2**: Model 1 + musculoskeletal disorders (osteoporosis, vertebral fracture, scoliosis, lower limb fracture, knee osteoarthritis, spinal canal stenosis, herniated disk, rheumatoid arthritis, and other musculoskeletal disorders or symptoms), cancer, and stroke. **Model 3**: Model 2 + occupation, type of employment, job time, shift system, status in employment, work-related physical activity (sitting time, standing time, walking time, strenuous work time), sedentary breaks during work time, screen time (TV viewing and smartphone, tablets, and PC use), physical activity over a week (walk, moderate-intensity, vigorous-intensity), sedentary time on holiday, muscle-strengthening activities, and pain. LS, locomotive syndrome; METs, metabolic equivalents; OR, odds ratio; PC, personal computer; Ref., reference; TV, television.

## 4. Discussion

This study reveals that among young and middle-aged Japanese workers, sedentary behaviors, including fewer sedentary breaks at work and prolonged TV viewing, were associated with LS, whereas M-PA was associated with a reduced risk of LS. These findings provide insights for preventing LS in this age group because even after middle age, it is important to emphasize exercise habits to improve the GLFS-25 score.^25^ Evaluating sedentary leisure time and work-related PA is crucial for preventing LS in young and middle-aged workers. Our findings bridge the knowledge gap in the association between sedentary behavior and LS.

A previous study reported that spending ≥28.01 minutes on moderate to vigorous PA (≥3.0 METs) was significantly associated with a lower risk of LS in older women (reference, ≤27.99 minutes; age-adjusted OR, 0.12),^14^ whereas resting and sitting behaviors (<1.5 METs) were not related to LS. Our study examined the associations between PA during leisure and work separately, accounting for potential confounders, and identified specific PA-related factors associated with LS in young to middle-aged participants. Our findings align with previous studies that revealed a relationship between PA and mobility. Elderly individuals who engaged in higher PA levels in midlife had better mobility.^26^ TV viewing time was identified as the most prevalent sedentary leisure activity and a key predictor of lower walking speed.^27^ Similarly, our findings indicate that sedentary behavior could be related to mobility, even in young or middle-aged individuals. Notably, fewer sedentary breaks and longer TV viewing times were associated with LS, independent of sitting time during work and sedentary time on holidays. Sedentary breaks were strongly associated with lower extremity function, independent of PA level or sedentary time.^28^ Although sedentary breaks during TV viewing and on holidays were not evaluated, longer sedentary bout duration may be a key factor in LS. Conversely, smartphone, tablet, and PC usage times were not significantly different between participants with and without LS and were not significantly associated with LS, which might not reflect sedentary behavior because of frequency and variations in usage patterns among individuals.

Participants with LS had a greater waist circumference and more dyslipidemia than those without LS but no differences in mean BMI. Sedentary behavior is related to dyslipidemia and lipid metabolism.^29^ Additionally, longer sedentary time and fewer sedentary breaks were associated with greater waist circumference.^30^ However, the abdominal circumference of LS participants was average, not pathological. Therefore, although the laboratory data on TG and HDL-C levels were not significantly different among the groups, a frequent history of dyslipidemia seemed characteristic of sedentary participants in this study.

In this study, 56.4% of participants with LS experienced body pain, which was higher than the prevalence of musculoskeletal disorders. Previous studies linked sedentary behavior with musculoskeletal pain.^31^ Among healthy office workers, active break intervals or postural shifts reduced neck and lower back pain.^32^ Although it was difficult to attribute body pain to prolonged sitting time, sedentary workers should be vigilant about musculoskeletal pain.

Over 90% of all participants were interested in their health; nevertheless, 44.5% of the participants had LS, and 76.5% of these had stage 1 LS (Table S1). Since this study included more participants with stage 1 LS who could walk independently, sedentary behavior might be associated with mild motor dysfunction in young and middle-aged workers. Screening for stage 1 LS is helpful for preventing motor disability in the future.^33^ Longitudinal studies have shown that LS stage 2 and above leads to an increased need for nursing care in the long-term care insurance system among middle-aged and elderly individuals.^8^^,^^9^ Therefore, early identification of stage 1 LS in young and middle-aged individuals is crucial to prevent progression to mobility dysfunction (LS stage 2 or 3).

This study had several limitations. First, PA was not evaluated using objective measurements, such as wearable accelerometer devices, which might have introduced questionnaire-related errors.^34^ However, both the IPAQ and WPAQ were verified using accelerometers. This method measured PA, including sedentary behavior during work and leisure time, among participants. Second, despite recruiting young and middle-aged participants, their engagement in M-PA and V-PA was low. Lifestyle changes, possibly due to the COVID-19 pandemic,^35^ may explain this trend, even though 2 years have passed since the pandemic occurred. It is noteworthy that, in this study, both M-PA and sedentary behavior were associated with LS. LS prevalence within each age group was higher than typically reported in nationwide epidemiological studies^9^^,^^10^ and approximately the same in each age group (Table S1), possibly because younger individuals concerned about their declining mobility participated. The response rate was 23.4% in this study, possibly due to the low awareness rate regarding LS among young and middle-aged workers. This underscores the importance of evaluating PA among individuals with LS in all age groups, including younger participants, for early detection and prevention of LS. Third, it is possible that there was some bidirectional causality, and the causal mechanisms linking LS to sedentary behavior were unclear in this study because of its cross-sectional design. Although LS could lead to reduced PA and increased sedentary time, all the participants were healthy workers, and a majority with LS had LS stage 1. Our findings suggest that maintaining mobility might be achieved through higher M-PA, more frequent sedentary breaks, and shorter TV screen time. Previous studies conducted on young and middle-aged office workers suggested that increased sedentary breaks were associated with reduced muscle inactivity.^36^ Reid et al^37^ found an association between TV viewing time and knee extensor strength, suggesting that addressing excessive sedentary time earlier in life may improve physical performance. Hence, reducing inactivity, enhancing muscle activity, and preserving muscle strength could aid in preventing LS and preserving mobility. Longitudinal studies are needed to investigate the association between LS and these factors. Although the causal relationship between PA and LS remains unknown, our results are consistent with previous observational studies showing that low PA in young and middle-aged individuals is associated with mobility disability in old age.^26^ Fourth, job-specific characteristics could not be evaluated, and categorizing participants into blue- or white-collar workers revealed no significant difference between those with and without LS, precluding adjusting for job type in the logistic regression analysis. However, an observational study suggested that sedentary behavior varies among individuals with the same occupation.^38^ Evaluating the job-specific characteristics of each occupation is essential to reveal the relationship between PA and LS based on occupation. Additionally, sex differences were not determined in this study because both men and women were assessed. Fifth, only the GLFS-25 was used to define LS without conducting additional LS tests. A previous study conducted among young and middle-aged workers reported that 23.1% of all participants aged 18-64 years had LS, and 18.3% of all participants were diagnosed as having LS by the GLFS-25.^39^ The GLFS-25 was able to determine LS better than the other LS tests—the 2-step test (prevalence of LS: 0.1% of all participants) and the stand-up test (prevalence of LS: 7.8% of all participants).^18^^,^^39^ Additionally, 39.1% of LS participants were under 45 years, and 14.6% of all participants under 30 years were diagnosed as having LS by the GLFS-25.^39^ Therefore, the GLFS-25 was appropriate to evaluate LS in young and middle-aged workers. For this study, the prevalence of LS was estimated to be about 18.0% from a previous report.^39^ The number of participants was considered sufficient to evaluate LS using multivariate analyses if approximately 50% of total participants returned the questionnaires. However, the prevalence of LS in this study was higher than that in the previous report.^39^ Therefore, there might have been sampling bias if more individuals who were concerned about their declining mobility participated in this study. The GLFS-25 showed characteristics as an indicator of incident functional disability.^8^ Furthermore, LS diagnosed using the GLFS-25 was associated with a lower maximum stride, slower usual gait speed, and longer timed up-and-go time; thus, the GLFS-25 is useful for assessing clinical mobility function.^17^ Japanese population-based cohort studies of elderly individuals demonstrated that increased M-PA and V-PA, and decreased sedentary time measured using self-reported questionnaires were associated with a lower risk of functional disability requiring nursing care.^40^ Therefore, our findings regarding the relationship between sedentary behavior and LS, defined by the GLFS-25, align with prior research exploring the relationship between PA and functional disability.

Despite these limitations, to the best of our knowledge, this is the first study to reveal the relationship between PA, sedentary behavior, and LS among young and middle-aged Japanese workers.

### 4.1. Conclusions

Our results suggest that increasing M-PA may reduce LS outcomes, whereas prolonged TV screen time and fewer sedentary breaks at work may be risk factors for LS in young and middle-aged Japanese workers. As a countermeasure against LS among young and middle-aged Japanese people, strategies to reduce sedentary time during leisure and work time are worth exploring.

## Supplementary Material

Web_Material_uiae001

## Data Availability

The datasets generated and/or analyzed during the current study are not publicly available because of a contract with the research cooperation institution, but are available from the corresponding author upon reasonable request.
