# Supplementary material for: Relationship between physical activity and locomotive syndrome among young and middle-aged Japanese workers
Source: J Occup Health. 2024 Jan 8;66(1):uiae001. doi: 10.1093/joccuh/uiae001 (PMC10878362; doi:10.1093/joccuh/uiae001)
Supplement: Web_Material_uiae001 [file web_material_uiae001.pdf]

Table S1. Prevalence of LS stage according to age groups

Age groups (ys)	non-LS<br>n = 186	LS stage 1<br>n = 114	LS stage 2<br>n = 22	LS stage 3<br>n = 13	*p*-value	Total<br>n = 335
**21-29**	22 (11.8)	13 (11.4)	1 (4.5)	2 (15.4)		38 (11.3)
**30-39**	55 (29.6)	26 (22.8)	5 (22.7)	4 (30.7)		90 (26.9)
**40-49**	42 (22.6)	32 (28.1)	9 (41.0)	2 (15.4)		85 (25.4)
**50-59**	46 (24.7)	27 (23.7)	6 (27.3)	3 (23.1)		82 (24.5)
**60-66**	21 (11.3)	16 (14.0)	1 (4.5)	2 (15.4)	0.802[a]	40 (11.9)

Notes: Data are presented as number (percentage).

a. examined by the chi-square test

Abbreviations: LS, locomotive syndrome; ys, years
